# The Effect of Aging on the Specialized Conducting System: A Telemetry ECG Study in Rats over a 6 Month Period

**DOI:** 10.1371/journal.pone.0112697

**Published:** 2014-11-14

**Authors:** Stefano Rossi, Ilaria Fortunati, Luca Carnevali, Silvana Baruffi, Francesca Mastorci, Mimosa Trombini, Andrea Sgoifo, Domenico Corradi, Sergio Callegari, Michele Miragoli, Emilio Macchi

**Affiliations:** 1 Department of Life Sciences, University of Parma, Parma, Italy; 2 Department of Neuroscience, University of Parma, Parma, Italy; 3 CERT, Center of Excellence for Toxicological Research, INAIL, ex ISPESL, University of Parma, Parma, Italy; 4 Department of Biomedical, Biotechnological and Translational Sciences (S.Bi.Bi.T.), Unit of Pathology, University of Parma, Parma, Italy; 5 Division of Cardiology, Fidenza Hospital, Fidenza, Italy; 6 Humanitas Clinical and Research Center, Rozzano, Milan, Italy; Brigham & Women's Hospital - Harvard Medical School, United States of America

## Abstract

Advanced age alone appears to be a risk factor for increased susceptibility to cardiac arrhythmias. We previously observed in the aged rat heart that sinus rhythm ventricular activation is delayed and characterized by abnormal epicardial patterns although conduction velocity is normal. While these findings relate to an advanced stage of aging, it is not yet known when and how ventricular electrical impairment originates and which is the underlying substrate. To address these points, we performed continuous telemetry ECG recordings in freely moving rats over a six-month period to monitor ECG waveform changes, heart rate variability and the incidence of cardiac arrhythmias. At the end of the study, we performed *in-vivo* multiple lead epicardial recordings and histopathology of cardiac tissue. We found that the duration of ECG waves and intervals gradually increased and heart rate variability gradually decreased with age. Moreover, the incidence of cardiac arrhythmias gradually increased, with atrial arrhythmias exceeding ventricular arrhythmias. Epicardial multiple lead recordings confirmed abnormalities in ventricular activation patterns, likely attributable to distal conducting system dysfunctions. Microscopic analysis of aged heart specimens revealed multifocal connective tissue deposition and perinuclear myocytolysis in the atria. Our results demonstrate that aging gradually modifies the terminal part of the specialized cardiac conducting system, creating a substrate for increased arrhythmogenesis. These findings may open new therapeutic options in the management of cardiac arrhythmias in the elderly population.

## Introduction

Population aging is of relevant concern since its exponential increment, due to lengthening of life, is strictly associated with rising hospitalization rates. Indeed, medical care related to cardiovascular morbidity, coronary diseases, atherosclerosis and hypertension, is reaching an epidemic proportion among the elderly. Cardiac aging is characterized by gradual, though distinct, alterations in myocardial structure and function. These changes must be distinguished from the pathological effects that occur for example in ischemic heart disease. Significant alterations of cardiac electrophysiological properties appear during the normal aging process of the ventricles as a result of structural remodeling of the extracellular matrix, modifications of cell-to-cell coupling between neighboring cardiomyocytes mediated by gap junctions, and changes in active membrane properties [1].

Cardiac aging in rats is characterized by a mild-to-moderate hypertrophy of the left ventricular mass [2] caused by an increase in myocyte volume [3]. Moreover, the number of cardiomyocytes is reduced as a consequence of necrosis and/or apoptosis. Such scenario is accompanied by an uncontrolled expression and production of fibronectin and collagen, which contribute to the expansion of extracellular matrix and collagenous septa [4].

Cardiomyocyte natural growth with age is associated with a structural and functional remodeling, which involves the spatial redistribution of electrical gap junctions. In the normal adult cardiomyocyte, gap junctions are found almost exclusively at the intercalated disks and are accordingly located at the end-to-end spatial coupling among the cardiomyocytes [5]. A structural mismatch of gap junctions is normally present between sinoatrial (SA) node and atria [6] and at the Purkinje-myocardial junctions [7]. Cellular distribution of gap junctions changes during normal ventricular growth, aging, and cardiovascular disease. Reduced intercellular coupling, due to age-related collagen deposition and decreased connexin (Cx) expression, particularly Cx40, might be responsible for delayed activation and conduction failure critically occurring at branching sites of the conducting system. At these divisions, source-sink mismatches may occur because of a change in the 3D architecture geometry affecting impulse propagation [8], [9].

In regards to cardiac cellular electrophysiology, a prolongation of the action potential (AP), an increase in cytosolic Ca^2+^ as well as a prolongation of the contraction phase have been observed [10]. Particularly it has been noticed that AP repolarization phase is highly prolonged in left ventricular cardiomyocytes isolated from the senescent compared to the young rat's heart [11]–[13].

Furthermore, the natural aging process is accompanied by a complex series of changes in the autonomic control of the cardiovascular system, favoring heightened cardiac sympathetic tone with parasympathetic withdrawal and blunted cardiovagal baroreflex sensitivity [14].

We previously observed in the aged rat heart that sinus rhythm (SR) ventricular activation was delayed and characterized by abnormal epicardial patterns while ventricular conduction velocity was normal [15]. Based on these observations, we hypothesized that the aging process is associated with changes in the distal conducting system, leading to uncoordinated and delayed myocardial excitation and contraction. Impaired interaction between the distal conducting system and the ventricular myocardium might create a potential reentry substrate, which would alter electrical stability and contribute to a higher incidence of ventricular arrhythmias in the elderly population [16], [17].

While these findings seem to characterize an advanced stage of cardiac aging, it is not yet known when and where the electrical impairment originates and how it evolves during senescence. Thus, the aim of the present study was to perform ECG radiotelemetry recordings in freely moving rats over a six-month period, in order to continuously monitor initiation and degree of ECG changes and the incidence of spontaneous arrhythmias. Our findings indicate that specialized conducting system alterations appear gradually over time during senescence, accounting for conduction impairment and increase in the incidence of arrhythmic events. These results open a new perspective to evaluate specialized conducting system performance and vulnerability for cardiac arrhythmias in aging population.

## Materials and Methods

### Ethics statement and animals

Experimental procedures and protocols were approved by the Veterinarian Animal Care and Use Committee of Parma University, with animals cared for in accordance with the European Community Council Directives of September 22, 2010 (2010/63/UE).

In this study, we used male Wild-type Groningen rats (Rattus norvegicus). This rat population was originally derived from the University of Groningen (the Netherlands), and it is currently bred in our laboratory under conventional conditions. Animals were kept at ambient temperature of 22±2°C and on a reversed 12:12 light-dark cycle (light on at 07:00 pm), with food and water available *ad libitum*. In this colony, 50% mortality occurs between 22 and 24 months of age.

### Radiotelemetry system, ECG recordings and analysis

Twenty-one animals of 18 month of age weighing 585±72 g were implanted with a radiotelemetry transmitter and monitored during the following six-month period. Only 7 animals survived until the 24^th^ month of age, which represents the end of the study. Thus, for homogeneity in statistics 7 animals of 12 months of age were considered as the control group in the present study.

The radiotelemetry transmitters utilized (TA11CTA-F40, Data Sciences International, St. Paul, MN, USA) were implanted according to a procedure described by Sgoifo and colleagues [18], which allows bipolar lead ECG recordings corresponding to vectorcardiographic Y lead (craniocaudal direction). Briefly, the transmitter body was placed in the abdominal cavity, one electrode (non-inverting) was fixed to the dorsal surface of the xyphoid process, and the other electrode (inverting) was placed in the anterior mediastinum close to the right atrium. Such electrode locations guarantee high-quality ECG recordings, even during vigorous physical activity. Immediately after surgery, rats were individually housed and injected for 2 days with gentamicin sulfate (Aagent 0.2 ml/kg, s.c., Fatro spa, Ozzano Emilia, Italy), and allowed 30 days of recovery before the start of ECG recordings. ECG signals were picked up by a radiotelemetry receiver (RPC-1) placed under the housing cage and recorded via ART-Silver 1.10 data acquisition system (1 kHz sampling frequency, Data Sciences Int., St. Paul, MN, USA).

During the follow-up period, 15-minute continuous ECG recordings were performed in aging animals once a week, on the same day of the week, between 8 and 10 am. In control animals, only one 15-minute continuous ECG recording was performed at 12 months of age. ECG signals were analyzed off-line using custom made software, in order to measure:

- RR interval duration, the time interval between two consecutive R wave peaks;
- PQ interval, the time interval between the onset of P wave (atrial depolarization) and the onset of QRS complex (ventricular depolarization);
- QRS complex duration;
- QT interval, the time interval between the onset of QRS complex and the end of T wave (ventricular repolarization);
- QTc interval, heart rate-corrected QT interval (Bazett's formula);
- frequency domain indexes of heart rate variability (HRV), computed by the fast Fourier transform, were obtained to gain information on sympathetic and parasympathetic modulation of heart rate. We considered the total power of the spectrum (ms^2^) and the power of the low frequency (LF; 0.2–0.75 Hz) and high frequency (HF; 0.75–2.5 Hz) bands in absolute values (ms^2^). The power of LF band is a non-specific index as it contains contributions of both the sympathetic and parasympathetic influences [19]; the power of HF band represents the activity of the parasympathetic nervous system and includes respiration-linked oscillations of heart rate [20]. The low frequency/high frequency ratio (LF/HF) estimates the fractional distribution of power and is taken as a synthetic measure of sympathovagal balance;
- number of arrhythmic events.

### Epicardial potential mapping

Epicardial potential mapping was performed in seven senescent rats (24 months of age) which completed the 6-month telemetry ECG recording period and in seven control animals (12 months of age). The animals were anesthetized with 0.015 ml/100 g body weight (BW) of Domitor (Orion Corporation, Espoo, Finland) and 0.04 ml/100 g BW of Imalgene (Merial, Tolouse, France). Additional amounts of anesthetic were provided during the experiment if needed. Under artificial respiration (Rodent Ventilator 7025, Ugo Basile, Comerio, Italy) the heart was exposed through a medial sternotomy. Body temperature was maintained constant at 37°C with infrared lamp radiation. During the experiment, the exposed heart and thorax were covered with a plastic film to maintain a moist and constant temperature environment. The high-density epicardial electrode array used in this study was previously described [15]. Regular 8×8 row and column electrode arrays were assembled on a surgical gauze, with 1 mm square mesh, corresponding to the inter-electrode distance [15]. Each electrode was constructed by fastening a 60 μm diameter insulated silver wire at the intersection of two orthogonal filaments of the surgical gauze under a stereo microscope (Cobra, Vision Engineering, Surrey, England). After all knots were fastened, the array was held upside down under the microscope and silver wire insulation was removed from a limited area on the epicardial side of each loop by deposition of a stripping paste. Finally, each electrode was optimally chlorided, as previously described [21]. Each electrode was connected to the non inverting input of an AC coupled, variable gain, differential amplifier of a 256 channel mapping system [22]. All inverting amplifier inputs were connected to the same reference electrode positioned on the left hind leg of the animal, while the ground electrode was positioned on the right hind leg. Unipolar electrograms (EGs) were recorded with a bandwidth of 0.03–500 Hz, amplifier input impedance of 10^12^ Ω and sampling rate of 1 kHz per channel. No additional filtering was used on the signals in order to minimize EG distortion.

Data were displayed off-line as epicardial EGs and activation isochrone contour lines by using custom made software. In each experiment, the electrode array was usually positioned on the anterior ventricular surface of the heart. The duration of P wave, PQ segment, and QRS complex was measured from the root mean square signal (RMS) computed from the 8×8 EGs recorded by the electrode array. The PQ segment, defined as the time interval between the end of atrial activation (P wave) and the beginning of ventricular activation (QRS complex), is an index of AV conduction time.

### Cardiac histopathology

At the end of cardiac mapping, animals were euthanized and the epicardial position of the electrode array was identified by fiducial marks. Thereafter, the heart was rapidly removed from the chest, weighed, and fixed in 10% buffered formalin solution for 24–48 h. The atria and the ventricles were excised from the heart and embedded into a paraffin block. Each block was fully sectioned and 4 µm thick histological sections were obtained [15]. These sections were subsequently stained with hematoxylin and eosin for fiber direction and fibrosis analysis. Fibrosis was assessed both in atrial and in ventricular myocardium.

### Statistics

All values are reported as mean ± standard deviation and analyzed using ANOVA followed by Student's t-test for unpaired values (p<0.01).

## Results

### Telemetry ECG signals

#### QRS waveform shape

**Figure 1:**
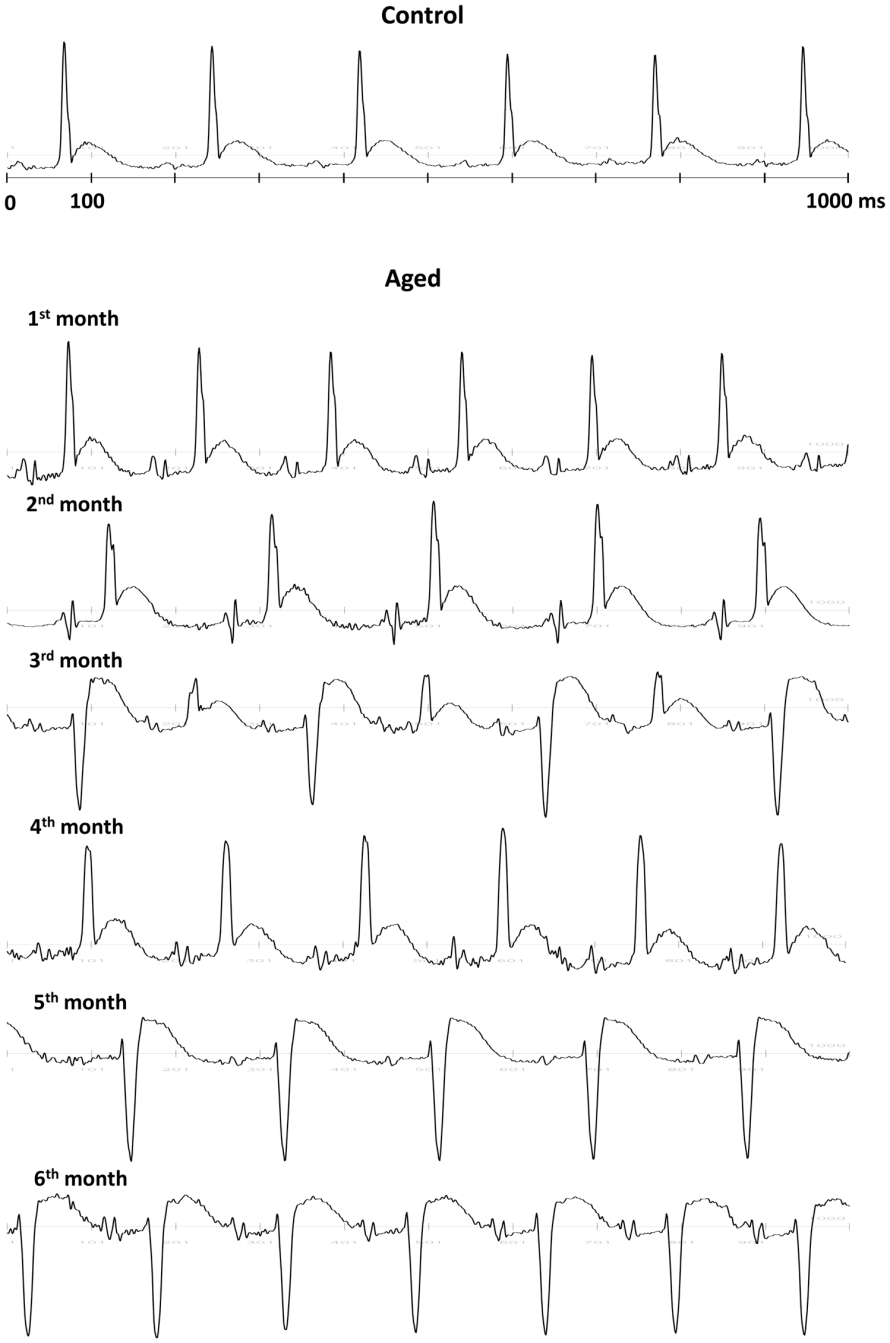
Telemetry ECG recordings. ECG traces (1 second) in a control animal (top) and in a representative aged animal displayed each month during the six-month study.

Telemetry ECG signals revealed gradual changes in waveform shape of the QRS complex during the six-month aging period. The QRS complex was initially positive (R-type) and became gradually negative (rS-type) in six out of seven animals. In only one animal the QRS complex remained always positive although its shape gradually changed. Figure 1 displays a representative ECG of a control animal and ECGs of an aged animal during each of the six-months showing gradual reversal in QRS polarity. Specifically, in the 3^rd^ month, the ECG displayed intervals in which the QRS complex was positive and intervals in which it became alternatively R-type and rS-type. In the 4^th^ month the QRS complex was again R-type while it turned rS-type during the last two months.

#### Waveform and interval durations

**Figure 2:**
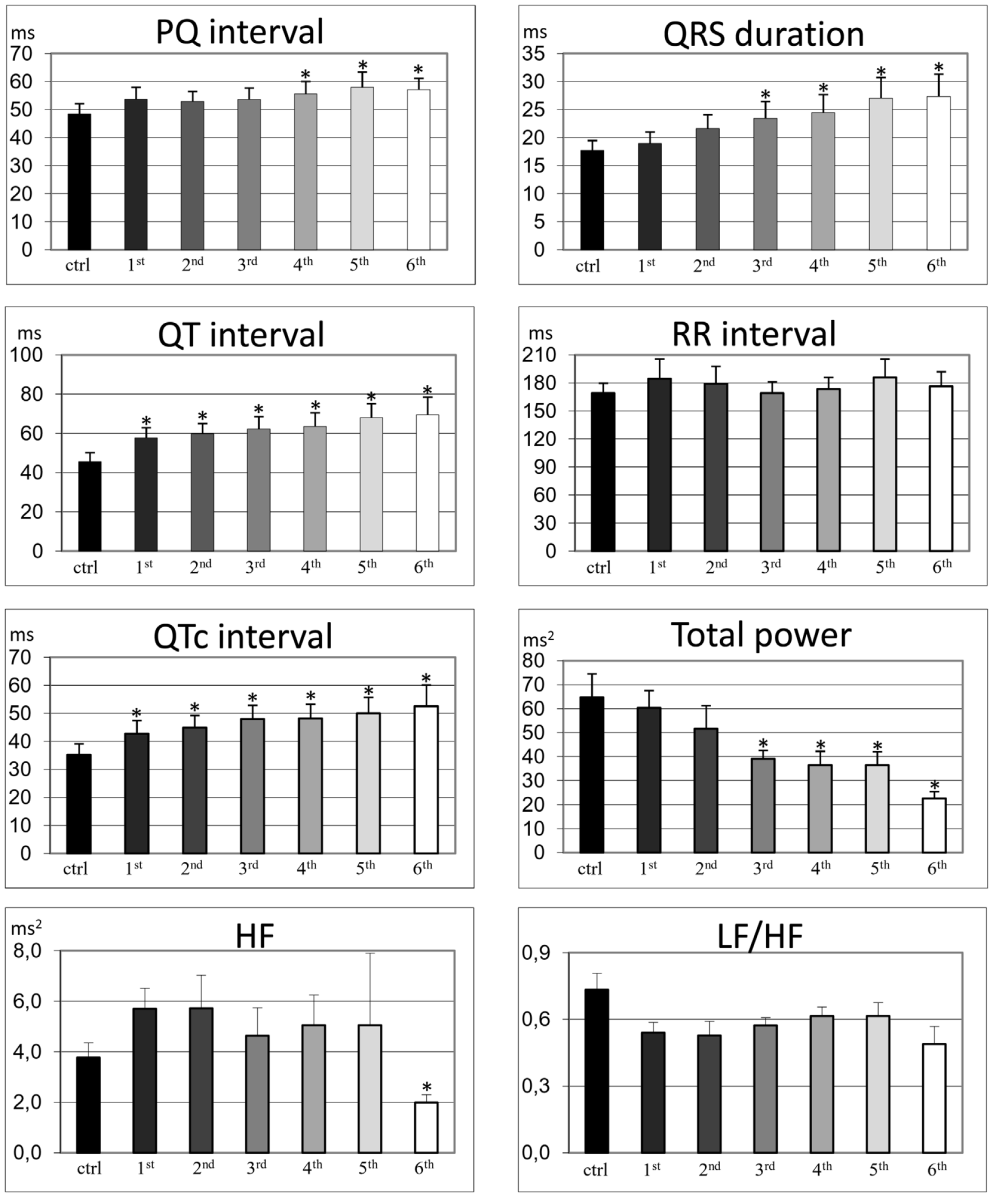
Telemetry ECG time and frequency domain analysis. Histograms of telemetry ECG basic electrophysiological parameters and frequency domain indexes of simpathovagal balance, evaluated from the 1^st^ to the 6^th^ month after radiotelemetry transmitter implantation of all animals. HF: High Frequency. LF: Low Frequency. * p<0.01 compared with control (ctrl).

Durations of the ECG waveforms and intervals measured during the six-months of aging were compared to controls (Fig. 2). The PQ interval showed an initial prolongation that became statistically significant only after the 4^th^ month; the total increase was 18% (ctrl: 48.5±3.4 ms vs. 6^th^ month: 57.1±4.1 ms). The QRS complex also showed a quasi-linear increase in duration which became significant only after the 3^rd^ month. In the 6^th^ month the increase of QRS complex duration was 53% (ctrl: 17.8±1.7 ms vs. 6^th^ month: 27.3±4.0 ms). The QT interval showed a gradual increase in duration, which became significant after the 1^st^ month. In the 6^th^ month the QT interval increase was 52% (ctrl: 45.6±4.5 ms vs. 6^th^ month: 69.4±9.1 ms). Heart rate did not change significantly during aging (ctrl: 169±11 ms vs. 6^th^ month: 176±16 ms) as reflected by similar changes in QTc interval (53% increase).

#### Heart rate variability

Analysis of heart rate variability revealed a gradual decrease in Total power values, which became statistically significant starting from the 3^rd^ month (Fig. 2). In the 6^th^ month the decrease was 65% (ctrl: 64.7±9.8 ms vs. 6^th^ month: 22.5±2.8 ms).

Similar values of HF spectral power were observed up to the 5^th^ month, while during the 6^th^ month HF values were significantly lower compared to control values. In the 6^th^ month the decrease was 47% (ctrl: 3.8±0.6 ms vs. 6^th^ month: 2.0±0.3 ms). No significant changes were observed in LF/HF ratio, in accordance with the absence of significant differences in RR intervals.

#### Incidence of arrhythmias

**Figure 3:**
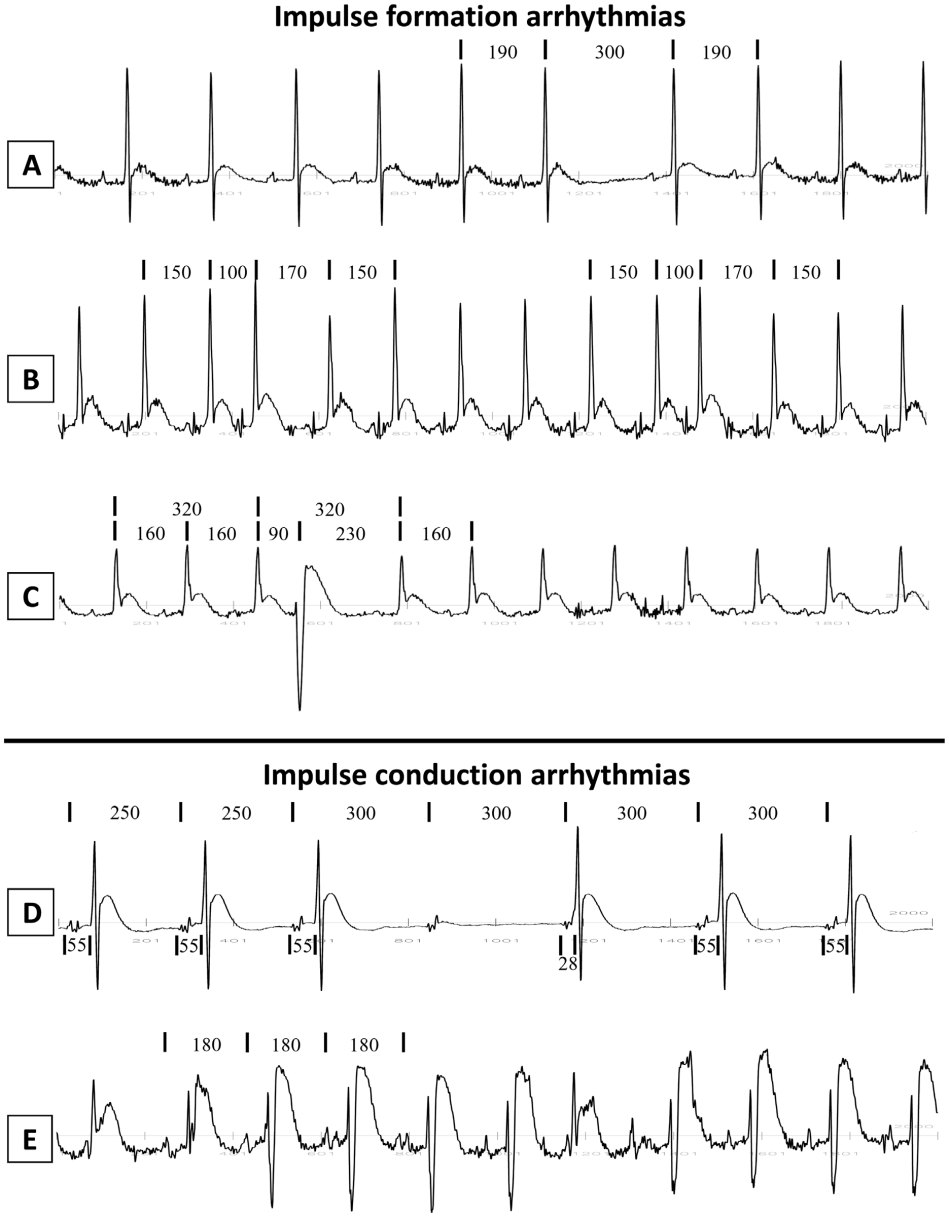
Impulse formation and conduction arrhythmias. Telemetry ECG recordings (2 seconds) of representative arrhythmic events, observed during the six-month period, that belong to the groups of impulse formation arrhythmia (A: sinus pause; B: atrial extrasystoles; C: ventricular extrasystole) and impulse conduction arrhythmia (D: second degree atrioventricular (AV) block with junctional escape beat; E: phasic aberrant ventricular conduction). Numbers identify time intervals in ms.

While spontaneous cardiac arrhythmias were absent in control rats, the incidence of arrhythmias increased during the six-months of aging. The different types of arrhythmia were classified according to their origin, as abnormalities in impulse formation or conduction [23] (Fig. 3).

1. Impulse formation arrhythmias: in this category we describe representative arrhythmic events of sinus pause, atrial extrasystole and ventricular extrasystole.
  - sinus pause. In Fig. 3A six sinus beats with RR interval of 190 ms were followed by a sinus beat with RR interval of 300 ms. This increase in cycle length represents a sinus pause because SR was restored during the following beats with the same RR interval of 190 ms.
  - atrial extrasystoles. Fig. 3B shows 3 sinus beats with an RR interval of ∼150 ms followed by a premature sinus beat with a coupling interval of 100 ms and a compensatory pause of 170 ms. The successive sinus beats resumed an RR interval of ∼150 ms. Thereafter, another premature sinus beat originated with a coupling interval of 100 ms and a compensatory pause of 170 ms. Again, the successive sinus beats resumed an RR interval of ∼150 ms.
  - ventricular extrasystole. In Fig. 3C three sinus beats with an RR interval of ∼160 ms were followed by a premature ventricular complex with a coupling interval of 90 ms. The QRS complex of the premature beat was not preceded by a P wave, had a negative waveform shape and longer QRS complex duration. The premature ventricular beat was followed by a compensatory pause of 230 ms. After 320 ms from the previous beat, normal SR was resumed with the same RR interval of ∼160 ms.
2. Impulse conduction arrhythmias: in this category we describe representative arrhythmic events of second degree atrioventricular (AV) block and phasic aberrant ventricular conduction.
  - second degree AV block. In Fig. 3D the first 3 beats were characterized by a PP interval of ∼250 ms and a PQ interval of ∼55 ms. The following P wave had a PP interval of 300 ms and was very likely characterized by a second degree AV block since it was not followed by a QRS complex. The following QRS complex represented a junctional escape beat because it was preceded by a P wave with a very short PQ interval (28 ms) and displayed the same shape and duration of the previous QRS complexes. All the following beats were characterized by PP intervals of ∼300 ms, PQ intervals of ∼55 ms and same QRS complex duration.
  - phasic aberrant ventricular conduction. In Fig. 3E the PP interval was ∼180 ms, but the QRS complex was characterized by two different morphologies. Specifically, three QRS complexes were R type while the other QRS complexes were rS type although with similar QRS complex duration. Isolated, bizarre QRS complexes are not always the result of ectopic ventricular discharges but may be due to temporary abnormal intraventricular conduction. To define these conditions more accurately, the term ‘phasic’ aberrant ventricular conduction is introduced to distinguish the temporary form from the ‘non-phasic’ or ‘permanent’ form of aberrant ventricular conduction [24].

**Figure 4:**
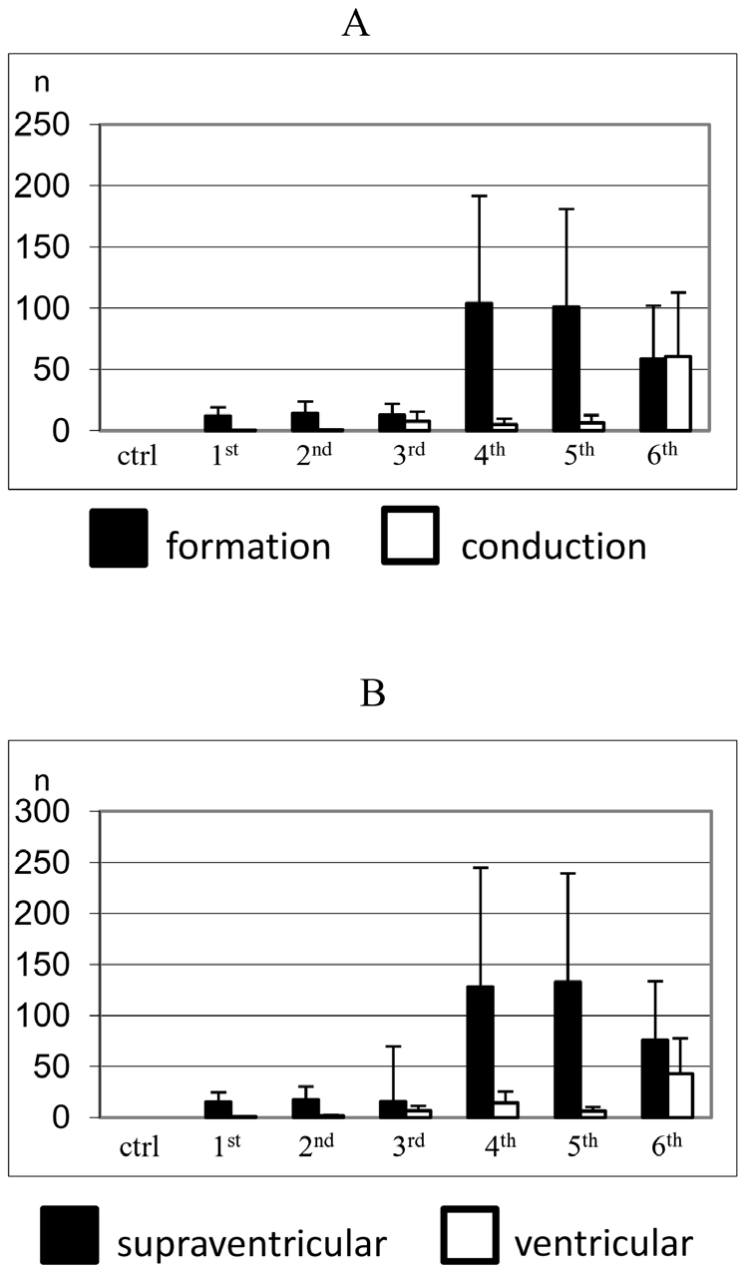
Arrhythmia type and location. Histograms relating to the type (A) and the location (B) of arrhythmic events of control and aged animals which occurred during telemetry ECG recording. Number of arrhythmic events was evaluated on the ECGs from the 1^st^ to the 6^th^ month after radiotelemetry transmitter implantation, as compared to controls (ctrl).

During the aging process the number and complexity of arrhythmic events increased, especially during the last three months of age. Fig. 4A shows, all the arrhythmic events that belong to abnormalities in impulse formation and abnormalities in impulse conduction at each month of telemetry recordings. The number of arrhythmic events increased with age, showing a peak in the 4^th^ month for abnormalities in impulse formation and a peak in the 6^th^ month for abnormalities in impulse conduction. On average, the total number of impulse formation arrhythmias largely exceeded the number of impulse conduction arrhythmias, during the six-month recording period.

All impulse formation and impulse conduction arrhythmias were also grouped according to their origin as supraventricular and ventricular arrhythmias. In agreement with this subdivision in Fig. 4B each bar represents supraventricular and ventricular arrhythmias during each month. It emerges that both supraventricular and ventricular arrhythmias increased from the 1^st^ month, with a peak in the 5^th^ month for supraventricular and in the 6^th^ month for ventricular arrhythmias. On average, the total number of supraventricular arrhythmias largely exceeded the total number of ventricular arrhythmias.

### Epicardial mapping

The RMS signal computed from the epicardial EGs revealed that during normal SR at advanced stage of age there was a significant increase in atrial activation time (control: 25.7±0.3 vs. aged: 32.9±0.2 ms, p<0.01), AV conduction time (control: 25.4±0.3 vs. aged: 36.6±0.3 ms, p<0.01) and total ventricular activation time (control: 18.8±0.1 vs. aged: 25.0±0.2 ms, p<0.01). Specifically, the increase corresponded to 28% for P wave, 44% for PQ segment and 33% for QRS complex duration.

**Figure 5:**
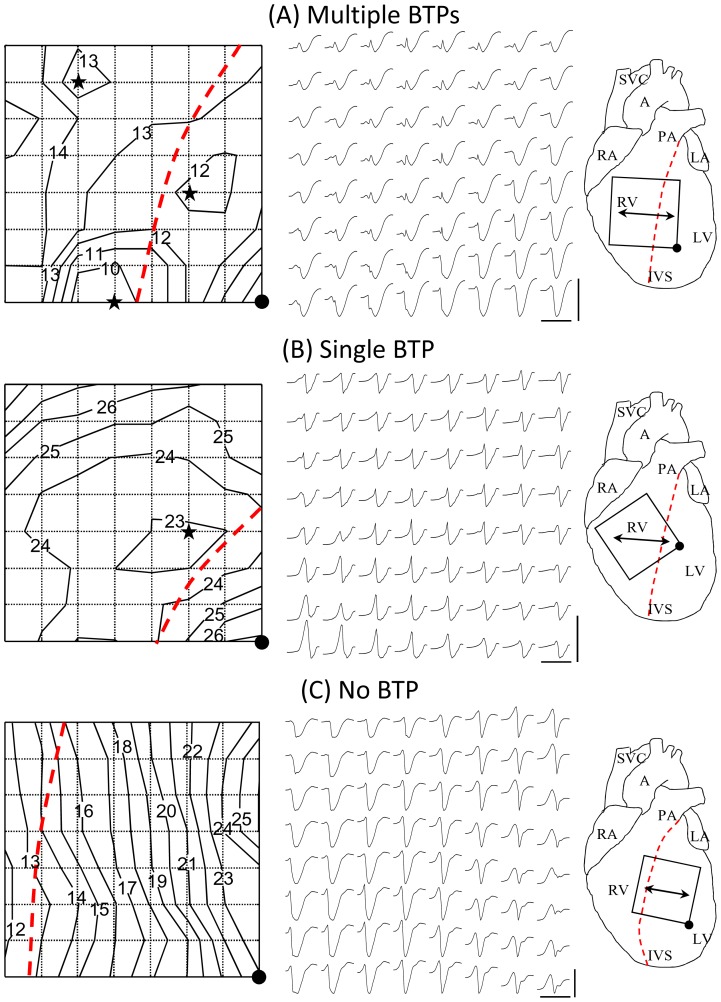
Sinus rhythm ventricular activations recorded in aged rats after the 6^th^ month. A: multiple breakthrough points (BTPs). B: single BTP. C: absence of BTP. *Left panels*: activation isochrone line maps. Numbers: activation times in ms from QRS onset; star: BTP site; dotted line, interventricular septum (IVS); black dot on the lower-right corner, reference point for epicardial orientation. *Middle panels*: 8×8 electrode array electrograms (EGs); vertical bar, maximum peak-to-peak EG amplitude (25 mV); horizontal bar, EG time scale (30 ms). *Right panels*: schematic view of the heart and epicardial electrode array position and orientation. PA, pulmonary artery; A, aorta; SVC, superior vena cava; RA, right atrial appendage; LA, left atrial appendage; RV, right ventricle; LV, left ventricle; dotted line, IVS; double arrowhead, epicardial fiber direction; square box, 8×8 electrode array with black dot for epicardial orientation (see above).

During normal SR different aberrant ventricular activation patterns (Fig. 5) were present, characterized by variable numbers of breakthrough points (BTPs) (detected in 4 animals), single BTP (detected in 1 animal) and absence of BTPs (detected in 2 animal).

#### Variable number of BTPs

Fig. 5A, left panel, shows ventricular activation patterns during normal SR characterized by 3 distinct BTPs emerging at 10, 12 and 13 ms from QRS onset, respectively. The location and number of BTPs gradually changed during successive beats, giving rise to variable activation patterns and wavefront collisions. The EG signals appeared to be prevalently negative in the regions of the earliest BKT. There was a small positive wave preceding the intrinsic deflection in regions of advancing wavefront towards the base of ventricles [25] (Fig. 5A, middle panel).

#### Single BTP

Fig. 5B, left panel, shows activation patterns with a single BTP which steadily occurred at 23 ms from QRS onset in the septal region of the RV. The elliptical isochrones about the BTP displayed mayor axis orientation counterclockwise (CCW) rotated from epicardial fiber direction, likely due to the endocardial site of impulse initiation and epi-endo CCW fiber rotation. Subsequently, the wavefront moved from the single BTP toward the apex and the base of the heart. The EGs were biphasic with a higher amplitude positive wave in the RV apex (Fig. 5B, middle panel).

#### Absence of BTPs

In the absence of BTPs in the measurement area, ventricular activation patterns were characterized by wavefronts that originated from distant areas outside the electrode array. In Fig. 5C, left panel, the isochrone map showed a planar wavefront originating from the RV and crossing the electrode array 12 ms from QRS onset. The wavefront travelled towards the LV with an apparent velocity of 0.5 m/s. The EGs were mostly negative (rS type) in the RV region proximal to the area of impulse initiation, and became mostly positive (Rs type) towards the free wall of the LV, in the region of advancing wavefront [25].

### Cardiac histopathology

**Figure 6:**
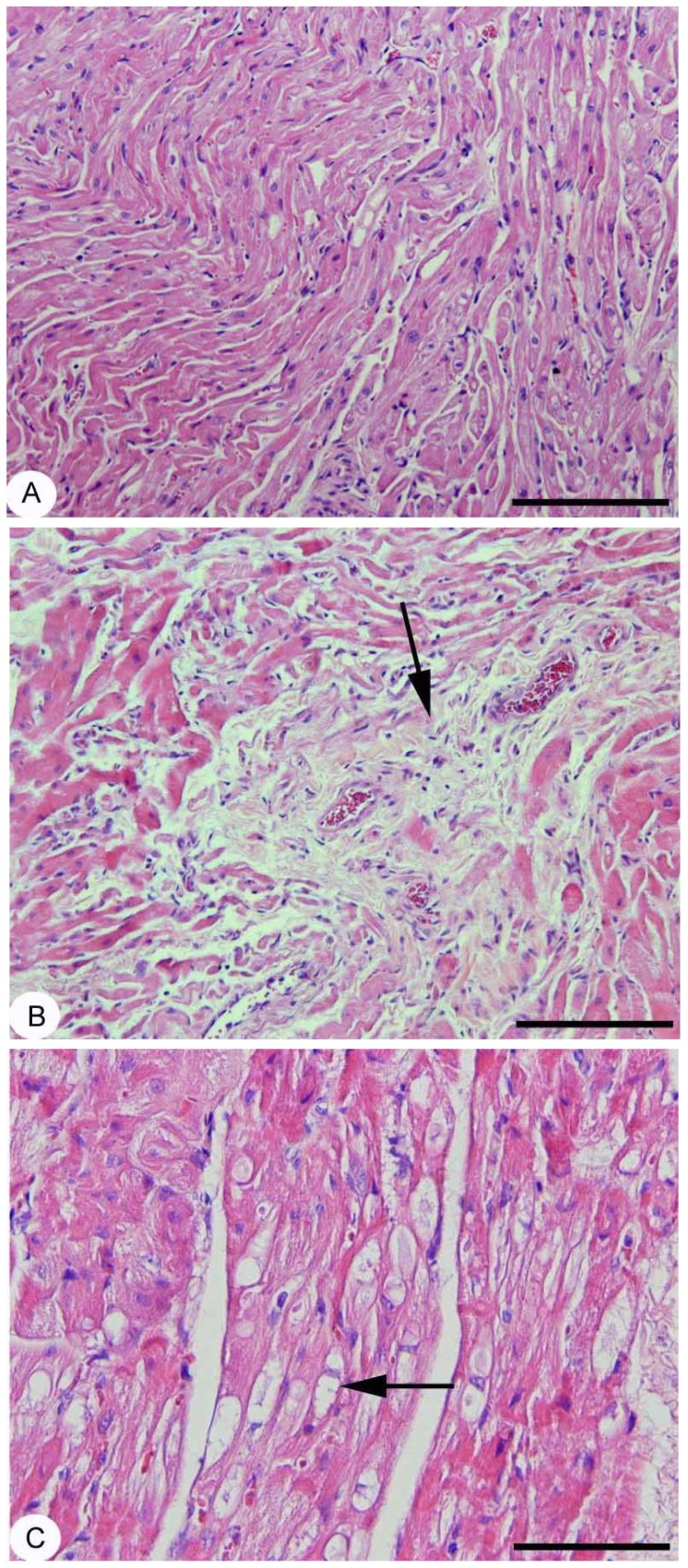
Atrial myocardial interstitial fibrosis. A: control heart section with no pathological interstitial collagen depositions; B: aged heart section with a focus of perivascular interstitial fibrosis (arrow); C: aged heart section with a focus of myocytolisis (arrow). Hematoxylin and Eosin staining. Original magnification: A and B ×10, C ×20 with scale bar in A and B 500 µm and in C 200 µm.

Microscopic analysis of atrial myocardium in control hearts revealed the presence of bundles of cardiomyocytes with various orientations. The inter-cardiomyocyte spaces were occupied by scanty connective tissue and microcirculation blood vessels usually running parallel to the major axis of the muscle fibers (Fig. 6A). Conversely, atrial myocardium in aged hearts was characterized bysignificant collagen deposition (Fig. 6B, arrow) and zonal phenomena of cardiomyocyte myocytolysis, i.e. perinuclear loss of sarcomeric elements resulting in an empty area (Fig. 6C, arrow).

Moreover, microscopic analysis of ventricular samples in control hearts did not show any sign of interstitial architectural remodeling, whereas in aged hearts the presence of scattered microfoci of interstitial myocardial fibrosis was revealed, in agreement with previous findings [15].

## Discussion

Our results indicate that aging gradually modifies impulse conduction through the terminal part of the specialized conducting system and ventricular myocardium, accounting for cardiac electrophysiological alterations during senescence and creating a substrate for increased arrhythmogenesis. Beside the impairment of the specialized conducting system, other concurring factors may contribute for the electrophysiological changes occurring with aging, such as the observed scattered microfoci of interstitial myocardial fibrosis and alterations in the cardiac autonomic control. The major findings supporting this conclusion are:

- progressive deterioration during telemetry ECG of ventricular activation revealed by i) gradual QRS increase in duration and change in shape and polarity; ii) increased vulnerability to arrhythmias with greater incidence of supraventricular than ventricular arrhythmias;

- loss of synchronization by the ventricular conducting system revealed by epicardial mapping;

- histopathological and 3D architectural disarrangement in aged myocardium.

### ECG waveforms and intervals duration changes

The analysis of telemetry ECG signals revealed that waveform and interval durations gradually increased with age, except for RR interval (Fig. 2). The PQ interval increased gradually, becoming 21% longer than control in the 6^th^ month of aging. The prolongation of PQ interval observed in our rats is likely due to conducting system impairment as previously demonstrated in rodents [11], [26]–[29] and humans [30]–[32].

In particular, during the 6-month period, the QRS complex gradually changed in shape from Rs to rS type (Fig. 1) and increased in duration (Fig. 2). In a recent study, Dupont et al. demonstrated that QRS morphology is a more important baseline ECG determinant of cardiac resynchronization therapy response than QRS duration alone [33]. Therefore, changes in QRS morphology should represent a marker of pivotal interest to detect changes in ventricular activation during the normal aging process.

In our previous study [15] we suggested that abnormal ventricular activation patterns recorded during SR in aged hearts may be due to the occurrence of impaired impulse conduction at the distal part of ventricular conducting system or at the junction between Purkinje fibers and ventricular myocardium [15]. If impulse conduction impairment had occurred at a proximal part of the ventricular conducting system, such as at a bundle branch or fascicle, the QRS waveform shape and duration would have changed abruptly and not gradually in time as it did in our study. Our findings suggest that impulse conduction impairment during aging initiated in a few discrete regions of the distal conducting system, and only at later stages gradually occurred in more diffuse regions ultimately affecting also the proximal conducting system.

The duration of the QRS complex increased by 55% compared to control animals (Fig. 2). The increase was gradual and became significant in the 3^rd^ month suggesting that impulse conduction impairment initiated in the distal conducting system. Such increase in QRS duration was also observed in other studies in rodents and it was ascribed to an increase in fibrosis and a reduction in conduction velocity across ventricular fiber direction [11], [27], [34]. Conversely, the limited deposition of fibrotic tissue and unchanged conduction velocity in the ventricular myocardium of our animals [15] suggests that QRS gradual increase in duration and change in shape are likely due to impulse conduction impairment in the distal conducting system.

The duration of QT interval increased gradually during the six-months of aging by 41% compared to control conditions (Fig. 2). The change in QT interval became statistically significant from the 1^st^ month suggesting that repolarization is continuously modulated by aging. Similar findings were also obtained in other rodent studies [26], [28] in agreement with the longer AP duration in older animals [12]. Prolonged QT interval was also associated with cardiac arrhythmia and sudden death in healthy people [35]. Heart rate did not change significantly during aging, while the increase in QTc interval was similar to the increase in QT interval.

### Cardiac autonomic control

Heart rate variability analysis was performed as a window into cardiac autonomic modulation. A reduction over time in the total variability of heart rate (as indexed by Total power values) was observed. Depression of heart rate variability, due to elevated sympathetic and/or reduced vagal tone, is a recognized risk factor for cardiac mortality [36], [37]. It is well known that in humans one of the most important features of the effects of aging on autonomic control of the heart is that vagal baroreflex sensitivity declines. Our results point to a clear decrease in vagal modulation of heart rate (as indexed by HF values) only during the final stage (the 6^th^ month) of the aging process. We can argue that the moderate, although not significant, increase in vagal tone observed during the initial stages was aimed at counterbalancing an increase in cardiac sympathetic influence on the SA node. This is supported by the fact that LF to HF ratio (index of sympathovagal balance) did not differ over time, suggesting that the regulatory influences of the vagal and sympathetic components on cardiac pacemaker activity were similarly balanced, leading to similar heart rate values. Further investigation is required in order to obtain a better understanding of the age-related changes in cardiac autonomic outflow in this rat model.

### Incidence of arrhythmias

This is, to the best of our knowledge, the first longitudinal study which reports the occurrence of different types of spontaneous arrhythmias in unrestrained telemetered animals during senescence, with the absence of any artificial means that could have favored the onset of arrhythmia [38]. Rhythm disturbances were observed during the aging process such as sinus pause, atrial and ventricular extrasystoles, AV block and phasic aberrant ventricular conduction. Quantitative analysis demonstrated that arrhythmias increased from the 1^st^ to the 6^th^ month with a greater incidence in the 4^th^ month, in a manner which reflected very closely prolongation of QRS complex duration and HRV reduction in the animals. The number of impulse formation arrhythmias exceeded four times of those of impulse conduction. Moreover, the number of supraventricular arrhythmias was five-fold greater than the number of ventricular arrhythmias. Importantly, these findings suggest that during aging the incidence of cardiac arrhythmias is greater in the atria than in the ventricles. This result is consistent with a reduction in the number of pacemaker cells in the SA node, atrial enlargement and increase in atrial fibrous and fatty tissue [6], [16]. Atrial tissue in our aged rats was characterized by an elevated degree in collagen deposition which involves an electrical remodeling. As a result, collagenous septa are formed acting as an obstacle to the normal impulse propagation, inducing a zig-zag pathway, slow conduction and finally unidirectional block increasing the susceptibility to structurally related supraventricular arrhythmias [39]–[42].

Moreover, the aged rat ventricular tissue displayed only the presence of scattered microfoci of interstitial myocardial fibrosis, which did not lead to decreased conduction velocity [15] and may explain the reduced number of ventricular arrhythmias observed in the present study.

### Epicardial ventricular activation patterns

The prolongation of epicardial EG waves and intervals duration in aged rats is in agreement with our previous results [15]. The increase in P wave duration is probably due to an increase in interstitial fibrosis with prolongation in the interatrial conduction time in aged rats [43]. The increase in PQ segment duration, index of an impairment in the AV conduction, is in agreement with previous findings in experimental animals [26] and humans [31]. Prolongation of QRS complex duration may be mainly ascribed to conduction impairment in the distal specialized conducting system. Indeed, epicardial mapping analysis in aged rats showed abnormal ventricular activation patterns which are also in agreement with our previous findings [15]. The abnormal activation is likely due to intercellular uncoupling between the specialized conducting system and the working myocardium. This alteration appeared gradually over time as demonstrated by the progressive telemetry QRS shape changes. Since in our aged rats ventricular tissue is characterized by small foci of subendocardial fibrosis, abnormal activation patterns are not due to fibrosis alone. Thus, the regions that may more easily undergo modification during the aging process and be responsible for delayed conduction are the ones that normally exhibit critical coupling like Purkinje-ventricular junctions [44].

### Histology

Structural alterations of cardiac tissue in our aged rats are in agreement with data reported in the literature [43]. Compared to controls, atrial myocardium in aged hearts was characterized by interstitial collagen deposition and myocytolisis, the latter being a phenomenon already described in the setting of the so-called hibernating myocardium and a part of atrial fibrillation structural remodeling [45]. An increase in P wave duration, an index of interatrial conduction delay, associated with atrial fibrosis may lead to enhanced vulnerability to atrial arrhythmia. This finding may explain why older animals reveal increased propensity to develop supraventricular arrhythmias [46]. Due to the absence of specialized conducting system in the atrial internodal myocardium, normal cardiac impulse is conducted throughout the atria and to the AV node by working cardiomyocytes. This impulse propagation follows well-defined atrial routes based on a non-uniform anisotropic conduction (i.e. strictly dependent on the anatomic orientation of the myocardial fibers). It may be hypothesized that any anatomic obstacle (e.g. interstitial fibrosis), in the way of impulse propagation, could imply increased propensity to develop supraventricular arrhythmias.

## Conclusions

In summary, we demonstrated that a longitudinal ECG study is essential for characterizing the progression of cardiac aging. Although our study is limited as an animal model, nevertheless it still suggests fresh therapeutic ramifications. For instance, in seeking to understand the likelihood of arrhythmias in elderly population, a frequent monitor of ECG characteristics may be of further importance for detecting temporal markers of cardiac electrophysiology and age-related events. While the aged-related risk stratification of arrhythmia vulnerability is still utopic, we stress the pivotal role of the specialized conducting system in the elderly since it could be fundamental in ensuing susceptibility of cardiac aging induced arrhythmias. Moreover, the results of the present study may offer insight into aging in humans and the possibility of early detection of age mediated deterioration of cardiac electrophysiology with the potential for early therapeutic intervention to retard that process.
